# The Loppered Milk Diet

**Published:** 1918-01

**Authors:** Geo. N. Jack

**Affiliations:** Buffalo


### The Loppered Milk Diet.*

### Including References to its Benefits in Post-Operative Disturbances and Metabolic Defectives.

DR. GEO. N. JACK, Buffalo.

(Continued from December issue.)

The seasons of the year in our climate that are most favorable for the unstable blood of the asthmatic, or that possesses more weather blood stablizing agents, are the spring summer solstice quadrant which extends approximately from May the 5th to Aug. the 11th, and the autumn winter solstice quadrant which extends approximately from Nov. the 6th to Feb. the 4th.

The seasons of the year that are the most unfavorable for the unstable blood of the asthmatic and the summer autumnal coryza victim, or that possess more weather blood disintegrating agents are the summer autumnal equinoctial quadrant which extends approximately from Aug. the 11th to Nov. the 6th, and the winter spring equinoctial quadrant which extends approximately from Feb. the 4th to May the 5th.

The summer autumnal equinoctial quadrant with its chilly, humid heavy dew, foggy, toxic ground gas nights, tends to disintegrate the unstable blood of the asthmatic and coryza subject.

These blood disintegrating summer autumnal nights are generally followed by hot, humid, windless, smoky atmospheric stasis days that cause a dilitation of the capillaries of the mucous membranes of the head, which favors a dumping of the disintegrated blood's waste products, producing the characteristic summer autumnal coryza.

The windless air stasis autumnal days also favor the accumulation of toxic blood disintegrating ground gases.

In some summer autumnal coryza subjects this blood dumping process does not extend in an incapacitating manner beyond the mucous membranes of the head, but not so in the asthmatic.

In the asthmatic the blood dumping process soon extends to the larynx where the ventricular bands are so thickened and hypertrophied by a dilitation of their mucous membrane capillaries and the accumulation of waste fluids from the disintegrating blood in the numerous wide lymph spaces found in them, that they project into the lumen of the larynx far enough to catch sufficient air on expiration to produce a valvular action that nearly closes the air tube. The process is also aggravated and the ventricular bands pushed farther into the lumen of the larynx by a great engorgement with dead leucocytes of the numerous mucous glands located in the ventricles of the larynx and their ascending pouches. This process nearly closes the common air tube at its laryngeal end, resulting clinically in a severe attack of asthma with a wheeze so loud that it often can be heard clear across the street. The loudness of the wheeze shows that it comes from the larynx and that it could not come from the smaller bronchi way down in the chest.

After the asthmatic recovers from the laryngeal involvement, by resolution and expectoration, the same process rapidly extends to the mucous membranes of the trachea and bronchi, narrowing and obstructing or plugging their lumen, producing attack after attack.

The dyspnoea with each air tube plugging process is aggravated by a dilitation and congestion of the dense capillary net-work of blood vessels found upon the walls of the air cells, thus rendering them erotic, which retards their normal expiratory partial collapse.

This abbreviated review of the pathology and physics of summer autumnal coryza and asthma as I have worked it out in previous papers shows the importance of the selection of a proper diet in these cases.

The prevailing weather conditions during the summer autumnal equinoctial quadrant, together with the gastro-intestinal disturbances common to that season of the year, produce more asthma than any other part of the year.

This summer and autumn have been so cold, windy, rainy and cloudy that there have been but comparatively few cases of summer autumnal coryza and asthma. Similar weather conditions prevailed in the summer and autumn of 1915, with a like decrease in the number of cases. The winds blew away the humidity and toxic ground gases and the rains also tended to wash the toxic gases from the atmosphere; and we had but few hot humid days to dilate the capillaries.

As soon as frosty or steady freezing weather sets in, the humidity is frozen up and the capillaries tightened up, which brings relief to the coryza subject and the vigorous asthmatic. The feeble asthmatic is made worse by the frosty air. The blood is driven from the surface capillaries to the deep organs, often so congesting the lungs as to terminate in bronchial pneumonia.

Summer autumnal coryza subjects and asthmatics are very sensitive to all dusts during attacks of coryza or asthma, but during favorable weather or seasons or when they are free from an attack, they, as a rule, are not affected by dust any more than normal people.

Most autumnal coryza subjects can during favorable weather, or the spring and winter months, work with a grain threshing machine, grain elevator, hay press or a grain mill where they come constantly in contact with air saturated almost to a suffocating point with pollen dust and other dust from every plant that grows without bringing on an attack of coryza or asthma or suffering any more inconvenience than a normal person would.

It would indicate in the case of summer autumnal coryza subjects and asthmatics that can work in pollen dust during favorable seasons without coryza or asthma, that their seasonal trouble is not due to a pollen dust anaphylaxis. Likewise it would indicate in the case of asthmatics that can eat all foods without asthma during favorable seasons that their asthma is not due to a food anaphylaxis.

The thick milk diet tends to add stability to the blood. I have seen people with an unstable hypersusceptible blood that was sensitized to numerous weather and food conditions, go through the blood disintegrating summer and winter equinoctial seasons while on a loppered milk diet without their usual seasonal or weather sickness.

After the blood has disintegrated from any cause, it has its vitality weakened, its metabolism shattered and is still further handicapped with an extraordinary blood dumping process. During this defensive period the blood is in no position to take on any more nourishment than is absolutely needed to maintain a spark of life and keep up the dumping battle and here again the loppered milk diet has often turned the battle tide and declared peace.

In post operative, metabolic or nutritional disturbances immediately following a serious operation where the nutrition of the patient is often a very delicate and most important problem, the loppered milk diet has often produced most gratifying results.

In post operative cases where the nutrition and metabolism are more or less permanently disturbed, as that following, cholecystectomy, gastro-enterostomy, thyroidectomy or ovariotomy, the loppered milk diet has often so successfully corrected the perversion as to permit the formation of fat to cushion in and take the pang and pull from the post operative adhesions. This fact has, I believe, in some cases prevented the development of a post operative wreck.

I now have under my observation four cases of chronic cholangeitis and cholecystitis, two of which have probable gall stone complications that have thrived and, up to date, gone from one to two years without any serious or incapacitating attacks while on the loppered milk diet and indicated medicinal treatment.

All of these four cases up to the time that they commenced treatment, had had frequent and serious attacks of liver and gall bladder troubles.

.Three of these four cases were confined in bed from two to three months before they commenced the loppered milk treatment and during these long attacks their liver gall bladder trouble was so serious together with the accompanying nausea, vomiting, chills, fever and emaciation that all operative procedures were postponed.

After partial recovery an operation was performed on one of these cases, a man aged sixty-six who had had periodic biliary attacks since sixteen years of age. We drained the gall bladder but did not find any gall stones.

The other three cases since their recovery from their last severe attacks are doing so nicely on the loppered milk diet in conjunction with indicated medicinal treatment that they have refused any operative interference.

My high blood pressure cases that have tried the loppered milk diet in conjunction with indicated medicinal treatment have obtained some very gratifying results.

I have come to base the prognosis of my functional anaemias and blood impoverishments upon the quantity of loppered milk that a given case will take.

In stomach diseases or gastro-intestinal disturbances there is no other diet that equals the loppered milk diet.

In impotency I have seen the loppered milk diet produce some surprising results, owing to the increased tone and nourishment of the vasomotors and capillaries. One case, a woman ten years married and the mother of four children, was cured of orgasmus impotency by the loppered milk diet, the first case of this character that I had seen cured. Some observers have claimed that about 40% of all women have orgasmus impotency, for which there is no cure, no matter whom they marry, the age at which they marry, nor the number or frequency of their pregnancies.

I have four cases of sugar diabetes that were cured of their emaciation, weakness, thirst, pruritis and general debility; and while they still show sugar in their urine they have led very active lives that required much physical exertion, without a breakdown, for from three to five years.

There is nothing better for kidney disturbances than the loppered milk diet and in many cases I have seen albumen disappear from the urine after a short trial of this diet.

Three cases of functional insanity that were cured under my observation by the loppered milk diet in conjunction with indicated medicinal treatment and spinal cupping, show that the loppered milk diet has a future in this large class of miserable individuals.

One patient, age seventeen, in charge of an attendant as she had attempted suicide, first consulted me Apr. 7, 1914. She conversed in a crying voice that was interrupted by frequent weeping spells. She had not menstrated for one and one-half years and was so anaemic that her blood penetrated test paper like pale ink; the red cells were granular, scant and 50% disintegrated. She was jaundiced with a large liver, much flatulency and frequent chills, resembling a hemolytic jaundice.

The result of the treatment was encouraging from the start. In seven days she came to the office alone and with a smile. This patient began with three pints of thick milk a day and at the end of four weeks was taking six pints a day, which amount she continued taking daily for two years. During these two years she gained forty pounds in weight. Patient had her first menstral show Sept. 10th and her first menstration Oct. 1st, or about six months after commencing treatment or two years after her last menstration. Menstrating has continued regular and normal since starting and the patient has remained well up to date.

One other patient I have had under observation only since July 5th or about four months but the result is very promising. When I first saw this patient she was so far advanced, so full of delusions, so excitable, unapproachable and apparently unmanageable that I advised her committal and accordingly called Dr. Nairn in consultation who agreed with me.

Before committing her however, at the request of her husband, we decided to try home treatment. Patient was a woman aged forty-nine, menopause age forty-two, never pregnant, weight 153 pounds, urine alkaline, loaded with phosphates, albumen more than a trace, indican blue.

This patient, in charge of an attendant, was put upon the loppered milk diet in conjunction with indicated medicinal treatment and sent out of town for three weeks, returning to Buffalo every week for inspection. After three weeks treatment patient was so much improved that she returned to her own home of which she took full charge.

This patient is still continuing her loppered milk diet, of which she averages about two and a half quarts a day and has continued to improve.

There is no diet for tuberculosis that has given me as satisfactory results as has the loppered milk diet. If my tubercular patients are taking six pints of milk a day, I assure them that they are on the best diet possible for their condition.

Gastro-intestinal flatulency is rarely met with in patients on the loppered milk diet. The antiseptic action of the lactic acid in the loppered milk prevents intestinal putrefaction, and its resulting toxic gases.

Loppered milk being fermented before it is taken acts as an anti ferment thus preventing the gases arising from intestinal fermentation. Loppered milk owing to its anti ferment and anti putrefactive action tends to prevent or overcome an acidosis which now has come to be recognized as one of the associates of physical degeneration or impending death.

The increased strength or endurance of people on a loppered milk diet is very marked. This one can understand by comparing the food value of milk with other foods. In this connection the Buffalo Sanitary Bulletin of May 31st, 1915, states that "The food value of one quart of milk is equal to either of the following: Three-quarters pound lean beef, two pounds of chicken, four-fifths pound loin of pork, three-fifths pound ham, three pounds fresh cod fish, one pint oysters, eight or nine eggs."

From an economic standpoint loppered milk is the cheapest possible diet one can live on even at eighteen cents per quart.

In about 90% of the cases in which I have prescribed the loppered milk diet, although at the first suggestion many gave a shudder of disgust, they have soon come to take it with a relish and to pronounce it the best thing they eat, the part of their diet that they always take with satisfying comfort and a feeling of immediate invigoration.

Loppered milk when properly prepared from rich, pure, clean, wholesome, raw milk, has a pleasant taste. If loppered milk is used before it has the proper age, it has a disagreeable acid taste. If milk is allowed to lopper in too warm a room or in an enclosed container, as bottles with caps on, it is apt to become lumpy when churned up and to have a rancid disagreeable taste; but when milk is properly loppered and served it constitutes a very palatable dish.

I do not advocate the loppered milk diet as a specific nor as a cure-all, but in conjunction with indicated medicinal or surgical treatment, I have seen it help out.

---

**Malingering by Garlic.** Geo. V. Perez, Med. Press, Dec. 20, 1916, states that inunctions are used in Spain to produce fever and that a garlic suppository produces a febrile reaction within 24 hours.

